# USP22 Suppresses *SPARC* Expression in Acute Colitis and Inflammation-Associated Colorectal Cancer

**DOI:** 10.3390/cancers13081817

**Published:** 2021-04-10

**Authors:** Robyn Laura Kosinsky, Dominik Saul, Christoph Ammer-Herrmenau, William A. Faubion, Albrecht Neesse, Steven A. Johnsen

**Affiliations:** 1 Division of Gastroenterology and Hepatology, Mayo Clinic, 200 First St SW, Rochester, MN 55905, USA; faubion.william@mayo.edu; 2 Department of General, Visceral and Pediatric Surgery, University Medical Center Goettingen, Robert-Koch-Str. 40, 37075 Goettingen, Germany; 3 Kogod Center on Aging and Division of Endocrinology, Mayo Clinic, 200 First St SW, Rochester, MN 55905, USA; Saul.Dominik@mayo.edu; 4 Department of Trauma, Orthopedics and Reconstructive Surgery, Georg-August-University Goettingen, 37075 Goettingen, Germany; 5 Department of Gastroenterology, Gastrointestinal Oncology and Endocrinology, University Medical Center Goettingen, Robert-Koch-Str. 40, 37075 Goettingen, Germany; christoph.herrmenau@med.uni-goettingen.de (C.A.-H.); albrecht.neesse@med.uni-goettingen.de (A.N.); 6 Gene Regulatory Mechanisms and Molecular Epigenetics Laboratory, Division of Gastroenterology and Hepatology, Mayo Clinic, Rochester, MN 55905, USA; Johnsen.Steven@mayo.edu

**Keywords:** inflammatory bowel diseases, colorectal cancer, USP22, H2Bub1, SPARC, epigenetics

## Abstract

**Simple Summary:**

Intestinal inflammation leads to an increased risk of developing colorectal cancer (CRC) and incidences are expected to rise. Therefore, it is crucial to identify molecular factors contributing to these medical conditions. In an earlier study, we identified USP22 as a tumor suppressor in CRC since the loss of *Usp22* resulted in severe tumor burden in mice. Moreover, *Usp22*-deficient mice displayed inflammation-associated symptoms. Therefore, we aimed to elucidate the function of USP22 in intestinal inflammation and inflammation-associated CRC. Indeed, mice with an intestine-specific loss of *Usp22* displayed more severe colitis compared to wild type controls. In addition, the loss of *Usp22* in a mouse model for CRC resulted in increased numbers of inflammation-associated tumors. Finally, we observed that the loss of USP22 induces the expression of *Sparc*, a factor previously linked to inflammation. Together, our results suggest that USP22 suppresses *Sparc* expression in acute colitis and inflammation-associated CRC.

**Abstract:**

As a member of the 11-gene “death-from-cancer” gene expression signature, ubiquitin-specific protease 22 (USP22) has been considered an oncogene in various human malignancies, including colorectal cancer (CRC). We recently identified an unexpected tumor-suppressive function of USP22 in CRC and detected intestinal inflammation after *Usp22* deletion in mice. We aimed to investigate the function of USP22 in intestinal inflammation as well as inflammation-associated CRC. We evaluated the effects of a conditional, intestine-specific knockout of *Usp22* during dextran sodium sulfate (DSS)-induced colitis and in a model for inflammation-associated CRC. Mice were analyzed phenotypically and histologically. Differentially regulated genes were identified in USP22-deficient human CRC cells and the occupancy of active histone markers was determined using chromatin immunoprecipitation. The knockout of *Usp22* increased inflammation-associated symptoms after DSS treatment locally and systemically. In addition, *Usp22* deletion resulted in increased inflammation-associated colorectal tumor growth. Mechanistically, USP22 depletion in human CRC cells induced a profound upregulation of secreted protein acidic and rich in cysteine (*SPARC*) by affecting H3K27ac and H2Bub1 occupancy on the *SPARC* gene. The induction of *SPARC* was confirmed in vivo in our intestinal *Usp22*-deficient mice. Together, our findings uncover that USP22 controls *SPARC* expression and inflammation intensity in colitis and CRC.

## 1. Introduction

Inflammatory bowel diseases (IBDs) affect one in approximately 250 Europeans and North Americans and incidences are expected to rise [1]. One possible complication in this group of diseases is the development of intestinal tumors and, notably, 15–20% of all cancer cases are correlated to pre-existing inflammation [2]. The fact that inflammation-associated colorectal cancer (CRC) was linked to particularly poor survival rates [3,4] underlines the need to identify key players involved in the development and progression of this disease.

Ubiquitin-specific protease 22 (USP22) was identified as a member of the so-called 11-gene “death-from-cancer” expression signature which was associated with distant metastasis, high recurrence rates and poor patient survival in several cancer entities, including CRC [5,6]. Indeed, the overexpression of USP22 in malignant diseases has been confirmed in numerous studies which were mainly based on immunohistochemical staining or mRNA quantification [7,8]. Accordingly, USP22 was considered a promising therapeutic target in cancer and efforts are underway to generate and optimize USP22 inhibitors [7].

USP22 is a ubiquitin hydrolase and, together with ataxin 7 (ATXN7), ataxin 7-like 3 (ATXN7L3) and enhancer of yellow 2 homolog (ENY2), builds the deubiquitinating module of the Spt-Ada-Gcn5 acetyltransferase (SAGA) complex. In addition, this transcriptional cofactor complex exerts histone acetyltransferase activity via general control nonderepressible 5 (GCN5) [9,10]. Within the SAGA complex, USP22 removes the monoubiquitination of H2B at lysine 120 (H2Bub1) which is associated with active gene expression [11]. Reduced H2Bub1 levels have been linked to advanced tumor grade and unfavorable outcome in CRC patients and, therefore, H2Bub1 has been described as a tumor-suppressive epigenetic mark [12]. In addition, USP22 was reported to deubiquitinate and thereby stabilize several non-histone substrates including tumor-promoting factors such as MYC [11]. 

In contrast to the general concept of USP22 being a universal oncogene, we and others uncovered a context-dependent tumor suppressor function of USP22 [8,13,14]. We demonstrated that the loss of USP22 resulted in increased proliferation and aggressive growth of human CRC cells. Moreover, USP22-deficient CRC cells displayed increased mTOR pathway activity as well as elevated sensitivity towards mTOR inhibition in vivo and in vitro. Notably, the intestine-specific deletion of *Usp22* in a genetic mouse model for adenomatous polyposis coli (APC)-associated CRC exacerbated the tumor phenotype, thereby confirming the context-dependent tumor-suppressive function of USP22. Interestingly, *Usp22*-deleted mice displayed symptoms associated with colitis such as weight loss, presence of occult blood, diarrhea and epithelial damage [13]. These findings suggest a potential role of USP22 in suppressing intestinal inflammation as well as inflammation-associated colorectal cancer.

In this study, we sought to investigate the consequences of an intestine-specific *Usp22* deletion in mice undergoing acute dextran sodium sulfate (DSS)-induced colitis as well as inflammation-associated CRC. Moreover, we aimed to decipher USP22-dependent molecular mechanisms using next-generation sequencing. Interestingly, we identified secreted protein acidic and rich in cysteine (*SPARC*), a factor which has previously been associated with intestinal inflammation, as a USP22 target in vitro and in vivo.

## 2. Materials and Methods

### 2.1. Animal Work

*Usp22*^flox^ mice were generated as described previously and were crossed with Villin-CreER^T2^ and *Apc*^1638N^ mouse lines (C57BL/6N background) to achieve a conditional, intestinal knockout in a genetic mouse model for CRC [13,15]. The *Apc*^1638N/+^ mouse line was a kind gift from F. Bosman (Erasmus University Medical Center Rotterdam, the Netherlands). Genotyping and knockout verification were performed as previously reported [13]. To induce the intestine-specific deletion of *Usp22*, mice were intraperitoneally injected with 1 mg tamoxifen (Sigma-Aldrich Co., St. Louis, MO, USA) per day for 5 consecutive days at the age of 4 weeks. To assess local and systemic effects of long-term colitis, 10-week-old animals were treated with low concentrations (0.75%) of dextran sodium sulfate (DSS; MO Biomedicals, LLC, Illkirch, France) for 14 consecutive days (*n* = 7 (Vehicle, *Usp22*^wt/wt^), *n* = 9 (Vehicle, *Usp22*^fl/fl^), *n* = 8 (DSS, *Usp22*^wt/wt^), *n* = 8 (DSS, *Usp22*^fl/fl^)) as described earlier [16]. To induce colitis-associated CRC, acute colitis was induced using high DSS concentrations (2% DSS for one week) in *Apc*-mutated animals (*n* = 16 (*Usp22*^wt/wt^), *n* = 13 (*Usp22*^wt/fl^), *n* = 9 (*Usp22*^fl/fl^)) at the age of 10 weeks. For health monitoring, the disease activity index (DAI) was determined [16]. The DAI was based on weight loss, stool consistency and intestinal bleeding intensity and was scored as follows: weight loss: 0–1% (0), 1–5% (1), 5–10% (2), 10–15% (3), >15% (4); stool consistency: normal (0), soft (1), very soft (2), diarrhea (3). The presence of occult blood was detected using the stool guaiac test by dissolving guaiac resin (Roth) in 70% EtOH until the solution was saturated. A few drops were applied to a Whatman filter paper and feces were distributed on the dried guaiac solution. Upon addition of 3% H_2_O_2_, blue staining was observed in the presence of blood which was scored as follows: no blue staining (0), weak/sporadic staining (1), medium staining (2), strong blue staining (3) and bloody anus (4). Upon sacrificing animals, blood was collected for subsequent serum analysis (Institute of Clinical Chemistry, University Medical Center Goettingen) and colons were flushed with PBS and cut longitudinally. Colon length was measured and tumor number and size were determined. Swiss rolls were prepared by rolling the colons upwards and colons were fixed in 4% formaldehyde in PBS overnight for subsequent paraffin embedding. Bones were snap-frozen in liquid nitrogen and stored at −80 °C until determining biomechanical parameters.

### 2.2. Histology and Determination of the Histo-Score (H-Score)

Hematoxylin and eosin (H&E) staining and immunohistochemical (IHC) staining of paraffin-embedded colons were performed as previously described [13,16]. For IHC, sections were blocked using 10% fetal bovine serum in PBS for 1 h and antibodies detecting CD45 (553089, BD Biosciences, Franklin Lakes, NJ, USA, 1:200) and SPARC (AF942, R&D Systems, Minneapolis, MN, USA, 1:200) were utilized. Biotinylated secondary antibodies (1:200; GE Healthcare, Chicago, IL, USA) and ExtrAvidin-Peroxidase (1:1000; Sigma-Aldrich) diluted in PBS were added, each for 1 h. Staining was developed using 3,3′-diaminobenzidin-tetrahydrochloride (DAB; Roth) and counterstaining was performed using hematoxylin. To quantify DAB staining intensity, five images were acquired per slide analyzed using FIJI (version 1.52). After deconvolution, DAB-containing images (8-bit images) were selected and the high-DAB area (threshold 115–255) and intensity were measured. Optical density was calculated by log(maximum intensity/mean intensity), whereby the maximum intensity was 255 for 8-bit images. Graphs were created and statistics were performed in R (version 3.6.3) with the following packages: ggplot2 3.3.2, dplyr 1.0.2, ggpubr 0.4.0, rstatix 0.6.0. Kruskal–Wallis and pairwise Wilcoxon rank sum tests were performed. To evaluate inflammation intensity, the degree of epithelial damage in the colon was determined on H&E-stained colon sections as previously described [16]. It ranged from 0 to 3, considering normal epithelium (0), as well as intestinal areas displaying mild (1), medium (2) or severe (3) epithelial damage.

### 2.3. Cell Culture and siRNA-Mediated Knockdowns

HCT116 human colorectal cancer cells were grown in McCoy’s medium supplemented with 10% fetal bovine serum, 100 units/mL penicillin and 100 μg/mL streptomycin at 37 °C and 5% CO_2_. siRNA (GE Dharmacon siGENOME, NT5: D-001210-05, siUSP22: MQ-006072-01) transfections using a non-targeting control (NT5) or targeting USP22 were performed using Lipofectamine^®^ RNAiMAX (Invitrogen, Carlsbad, CA, USA) according to the manufacturer’s instructions. Cells were harvested after 72 h.

### 2.4. Quantitative Real-Time PCR (qRT-PCR)

RNA extraction, reverse transcription and qRT-PCR were performed as previously described [17]. Briefly, RNA was isolated using TRIzol (Invitrogen) and reverse transcribed using Moloney murine leukemia virus reverse transcriptase (New England Biolabs, Ipswich, MA, USA) and random primers. Gene expression analysis was performed by qRT-PCR using SYBR Green I (Roche Diagnostics, Basel, Switzerland). All expression values were normalized to 18S rRNA levels. The following forward (F) and reverse (R) primers were used: *18SrRNA* F 5′-AACTGAGGCCATGATTAAGA-3′, R 5′-GGAACTACGACGGTATCTGA-3′; *USP22* F 5′-AGCCAAGGGTGTTGGTCGCG-3′, R 5′-ACTGCCACCACGCCCGAAAG-3′; *SPARC* F 5′-CTGCGGGTGAAGAAGATCCA-3′, R 5′-TGGGAGAGGTACCCGTCAAT-3′.

### 2.5. Western Blot

For Western blot analyses, control and USP22-depleted HCT116 cells were lysed in radioimmunoprecipitation assay (RIPA) buffer (1% NP-40, 0.5% sodium deoxycholate and 0.1% SDS in PBS) containing protease, phosphatase and deubiquitinase inhibitors (1 ng/μL aprotinin/leupeptin, 10 mM β-glycerophosphate, 1 mM N-ethylmaleimide, 1 mM Pefabloc). Upon sonication for 15 min, proteins were denatured in 6× Laemmli buffer at 95 °C for 5 min, separated by SDS-PAGE and blotted onto nitrocellulose membranes. The following antibodies were used: anti-H2B (ab1790, Abcam, Cambridge, UK, 1:5000), anti-H2Bub1 (5546, Cell Signaling, Danvers, MA, USA, 1:1000), anti-USP22 (sc-390585, Santa Cruz, Dallas, TX, USA, 1:1000).

### 2.6. mRNA-Seq and Analysis

Our previously published mRNA sequencing data (ArrayExpress; accession number: E-MTAB-7393) were aligned to the human reference genome GRCh38.p10 and analyzed as reported earlier [14]. A volcano plot was generated using R version 4.0.2 (EnhancedVolcano 1.8.0). Gene set enrichment analysis (GSEA) [18,19] was performed using custom gene sets containing genes significantly regulated in mice experiencing acute DSS-induced colitis [20] and in intestinal biopsies isolated from IBD patients [21,22].

### 2.7. Chromatin Immunoprecipitation (ChIP) and ChIP-qPCR

ChIP was performed as previously described [13,15]. H3K27ac (C15410196, Diagenode) and H2Bub1 (5546, Cell Signaling) occupancy was determined in HCT116 cells with a siRNA-mediated USP22 depletion as well as control cells (NT5). IgG (C15410206, Diagenode) was used as a negative control and samples were compared to inputs. Primer sequences for subsequent qPCR were as follows: H3K27ac *SPARC* pos. F 5′-ATGTCGATGTGGCAGCTGAT-3′, H3K27ac *SPARC* pos. R 5′-ATTAGAGGGCATGACGTGGG-3′; H3K27ac/H2Bub1 *SPARC* neg. F 5′-GAGAGACCACTTACCCGCAG-3′, H3K27ac/H2Bub1 *SPARC* neg. R 5′-TACAGGGTGACCAGGACGTT-3′; H2Bub1 *SPARC* pos. F 5′-GGCCCAAGGACACTCACATT-3′, H2Bub1 *SPARC* pos. R 5′-CCTTCGACTCTTCCTGCCAC-3′.

### 2.8. Bone Biomechanics

Biomechanical properties of tibiae were assessed using a Zwick device as described earlier [16]. After fixation on a plate, a primary force of 1 N was applied, and a stamper moved towards the bone with a speed of 50 mm/min. The resulting test accuracy was 0.2–0.4%, using 2–500 N. The measurements were aborted if the curve declined by 10 N. The applied strength during deformation (yield load), before (failure load) and during the fracture (Fmax) and the slope of the curve (stiffness) were determined using testXpert software (Zwick GmbH KG).

### 2.9. Statistics

Graphs were generated with GraphPad Prism version 9.0.0 (GraphPad Software, Inc., San Diego, CA, USA) as well as R version 3.6.3 or 4.0.2. Statistical analyses were performed using a *t*-test or one-way ANOVA and subsequent Tukey post hoc test (α = 0.05, * *p* ≤ 0.05, ** *p* ≤ 0.01, *** *p* ≤ 0.001, **** *p* ≤ 0.0001).

## 3. Results

### 3.1. Intestinal Usp22 Deletion Exacerbates Colitis Intensity in Mice

**Figure 1:**
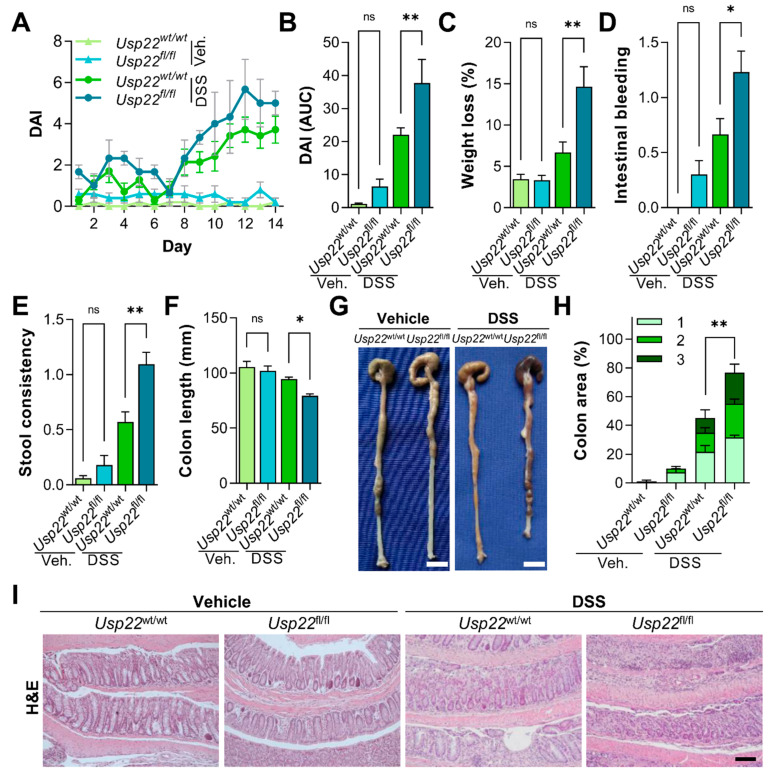
The intestinal deletion of ubiquitin-specific protease 22 (*Usp22)* exacerbates dextran sodium sulfate (DSS)-mediated colitis. (**A**) Villin-CreER^T2^, *Usp22*^flox^ mice were treated with 0.75% DSS or H_2_O alone (vehicle) for 14 days and sacrificed on day 15. The disease activity index (DAI) was determined daily and the (**B**) area under the curve was calculated. (C-F) The increased DAI was mediated by elevated weight loss (**C**), the presence of occult blood (**D**) and diarrhea (**E**) in DSS-treated *Usp22*^fl/fl^ mice. (**F**) The colon length of *Usp22* knockout animals during acute colitis was reduced. (**G**) Representative images of colons. Scale bar: 1 cm. (**H**) H&E-stained colon sections were analyzed and the percentage of epithelium displaying mild (1), moderate (2) and severe (3) damage was determined. *Usp22* deletion exacerbated colitis-associated tissue damage. Statistical analyses are based on the H-score. (**I**) Representative images of H&E-stained colon sections. Scale bar: 100 µm. Mean ± SEM. One-way ANOVA. * *p* ≤ 0.05, ** *p* ≤ 0.01.

We previously demonstrated that the intestine-specific knockout of *Usp22* results in colorectal epithelial damage in mice [13]. In agreement with these findings, a Villin-CreER^T2^-driven *Usp22* deletion alone resulted in a slightly increased colitis-related disease activity index (DAI; Figure 1A,B) which was calculated based on weight loss, intestinal bleeding intensity and stool consistency (Figure 1C–E). Thus, we sought to test the consequences of an intestinal *Usp22* deletion in acute dextran sodium sulfate (DSS)-induced colitis. Interestingly, DSS-treated Villin-CreER^T2^, *Usp22*^fl/fl^ mice displayed increased weight loss, intestinal bleeding and a higher incidence of diarrhea. In addition, these animals had significantly shortened colons, supporting the hypothesis that the loss of *Usp22* elevates colitis severity (Figure 1F,G). Next, we evaluated the degree of inflammation-associated intestinal epithelial damage and, indeed, DSS-treated *Usp22* knockout animals displayed a higher degree of moderate and severe epithelial damage compared to wild type littermates (Figure 1H,I). Together, these findings demonstrate that the loss of *Usp22* in a mouse model for colitis significantly elevates inflammation-associated signs.

### 3.2. Usp22 Deficiency Increases Local and Systemic Inflammation after DSS Treatment

**Figure 2:**
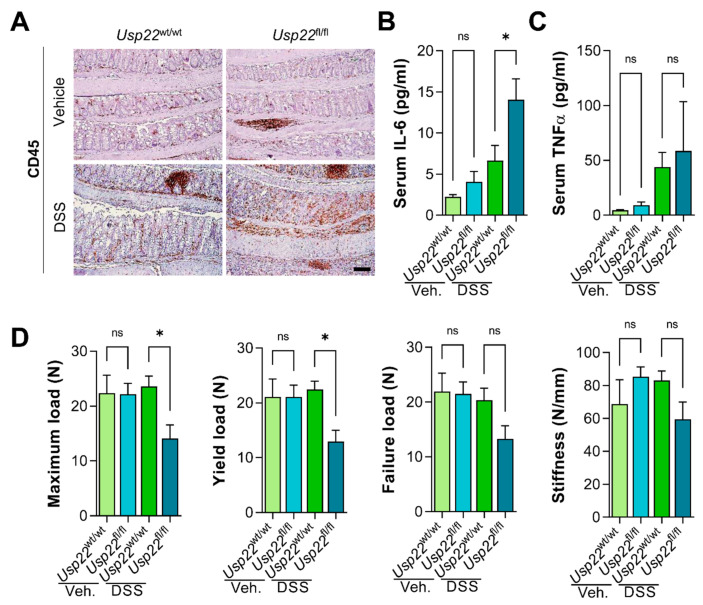
Local immune cell infiltration and systemic inflammation were elevated in *Usp22*-deficient mice. (**A**) Immunohistochemistry (IHC) for CD45-positive cells in the colon revealed increased infiltration of immunoregulatory cells in DSS-treated Villin-Cre, *Usp22*^fl/fl^ mice. Scale bar: 100 µm. (**B**) *Usp22*-deficient mice displayed higher serum interleukin 6 (IL-6) levels while (**C**) serum TNFα levels were not significantly affected by genotype. (**D**) Tibia biomechanical properties were determined using a Zwick device. Maximum, yield and failure load as well as bone stiffness were decreased in DSS-treated *Usp22*^fl/fl^ mice, indicating higher bone fragility in these animals. Mean ± SEM. One-way ANOVA. * *p* ≤ 0.05.

The observation that *Usp22*^fl/fl^ mice displayed more severe colitis-associated symptoms was supported by an increase in CD45-positive immune regulatory cells in the colon of these animals after DSS treatment (Figure 2A). Besides signs of local inflammation, we evaluated whether *Usp22* deficiency was associated with increased systemic inflammation. While the colitis-associated increase in serum tumor necrosis factor α (TNFα) levels was not exacerbated among genotypes, serum interleukin 6 (IL-6) levels were significantly increased in *Usp22^fl^*^/fl^ animals undergoing acute colitis (Figure 2B,C). To further examine the systemic inflammatory events, we evaluated bone biomechanical properties since IBD patients as well as mice undergoing colitis display decreased bone mineral density and increased fracture risk [23,24,25]. Indeed, the forces required to induce bone bending and fracture were significantly lower in animals with intestine-specific *Usp22* loss compared to their wild type littermates, indicating higher bone fragility in *Usp22*^fl/fl^ mice (Figure 2D). In summary, the intestinal deletion of *Usp22* results in both increased local and systemic inflammation.

### 3.3. Usp22 Deficiency Decreases Survival and Promotes Inflammation in a Murine Model of Inflammation-Associated CRC

**Figure 3:**
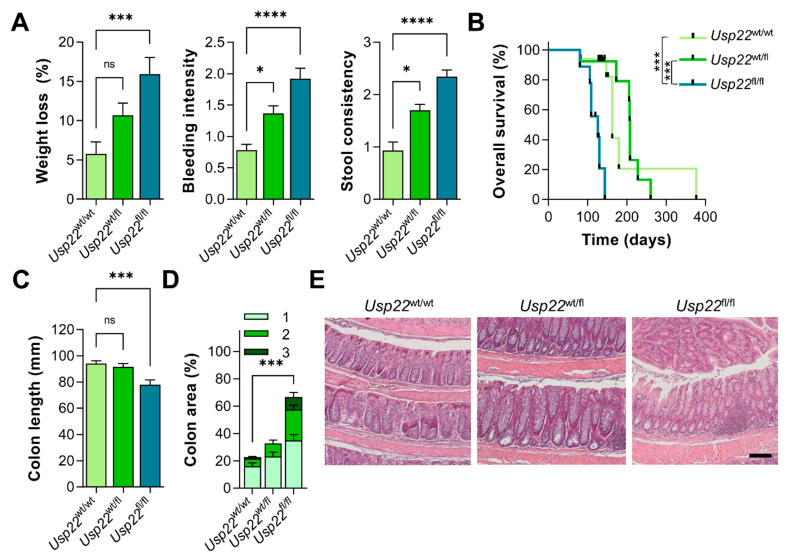
Inflammation-associated symptoms were aggravated in *Apc*-mutant *Usp22*^fl/fl^ mice. (**A**) Average weight loss, scores for bleeding intensity and stool consistency were significantly increased in *Apc*^1638N/+^, Villin-CreER^T2^, *Usp22*^fl/fl^ mice compared to *Usp22* wild type controls. (**B**) These *Usp22*-deficient animals displayed reduced overall survival. (**C**) To assess phenotypic and histological features, wild type and heterozygous controls were sacrificed at the same age that *Usp22*-deficient mice were sacrificed. Consistent with a higher disease activity, *Usp22*^fl/fl^ mice displayed decreased colon lengths. (**D**) Analysis of H&E-staining of colon sections revealed elevated inflammation-associated epithelial damage in *Usp22*^fl/fl^ mice. Statistical analyses are based on the H-score. (**E**) Representative images of H&E-stained colon sections. Scale bar: 100 µm. Mean ± SEM. One-way ANOVA. * *p* ≤ 0.05, *** *p* ≤ 0.001, **** *p* ≤ 0.0001.

Based on the finding that 15–20% of all cancer cases are associated with pre-existing inflammation and there is a strong link between intestinal inflammation and colorectal cancer [2,3], we sought to elucidate whether mice with an intestinal deletion of *Usp22* were more prone to developing inflammation-associated CRC. For this purpose, we combined intestinal deletion of *Usp22* with a mouse model containing a mutated *adenomatous polyposis coli* (*Apc*) and treated animals with DSS for one week to induce intestinal inflammation. The disease activity was evaluated regularly and, as hypothesized, *Apc*^1638N/+^, Villin-CreER^T2^, *Usp22*^fl/fl^ mice displayed severe weight loss, intestinal bleeding and diarrhea (Figure 3A). Accordingly, the survival of *Usp22*-deficient animals was significantly decreased (Figure 3B). As expected, these animals displayed shortened colons as well as severe intestinal epithelial damage (Figure 3C–E). These findings suggest that the intestine-specific deletion of *Usp22* in the background of an *Apc* mutation is associated with increased inflammation and early lethality.

### 3.4. Usp22 Deficiency Promotes Inflammation-Associated CRC

**Figure 4:**
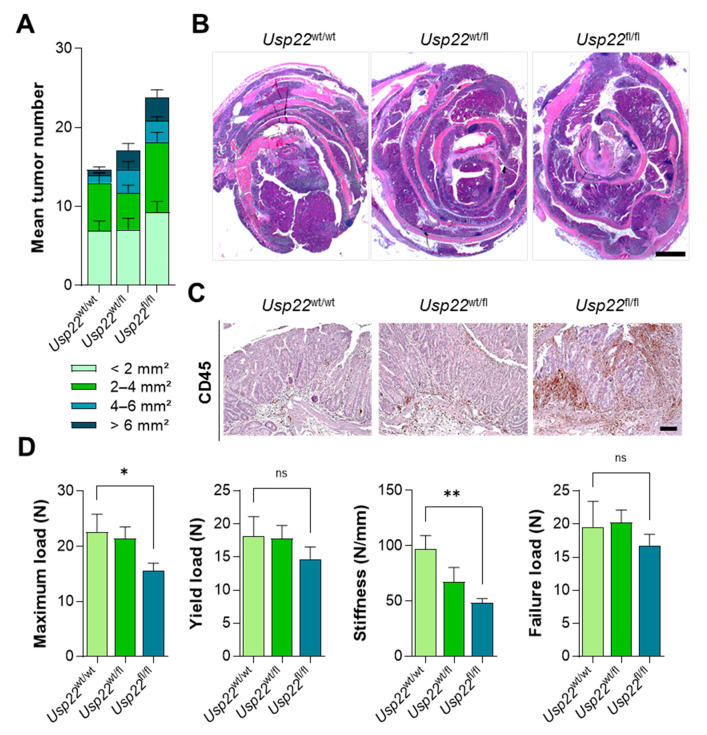
Colorectal tumor burden is increased in *Apc*-mutant mice with an intestinal *Usp22* deletion. (**A**) In Villin-CreER^T2^ mice carrying an *Apc*^1638N/+^ mutation, a conditional *Usp22* deletion led to increased tumor number and size following DSS treatment. (**B**) Representative images of H&E-stained colons isolated from *Apc*^1638N/+^, Villin-CreER^T2^, *Usp22*^flox^ mice. Scale bar: 1 mm. (**C**) IHC revealed increased infiltration of CD45-positive cells into colorectal tumors of *Usp22*-deleted mice. Scale bar: 100 µm. (**D**) Increased bone fragility was detected in *Apc*^1638N/+^, Villin-CreER^T2^, *Usp22*^fl/fl^ mice using biomechanical analyses of tibiae. Mean ± SEM. One-way ANOVA. * *p* ≤ 0.05, ** *p* ≤ 0.01.

Besides inflammation-associated colorectal epithelial damage, we detected an increased tumor number as well as size in *Apc*-mutated *Usp22*^fl/fl^ mice compared to *Usp22* wild type litter mates (Figure 4A,B). In agreement with the observation that these mice displayed increased inflammation, the number of CD45-positive immune regulatory cells was elevated in tumors of *Usp22*^fl/fl^ animals (Figure 4C). Finally, bone biomechanical properties were assessed and, as expected, tibiae isolated from *Apc*^1638N/+^, Villin-CreER^T2^, *Usp22*^fl/fl^ mice were more fragile compared to control animals (Figure 4D). In summary, *Apc*-mutated mice with an intestinal deletion of *Usp22* displayed increased colorectal tumor burden, immune cell infiltration and increased bone fragility.

### 3.5. USP22 Depletion Promotes IBD-Associated Gene Expression Programs and SPARC Expression in Human CRC Cells

**Figure 5:**
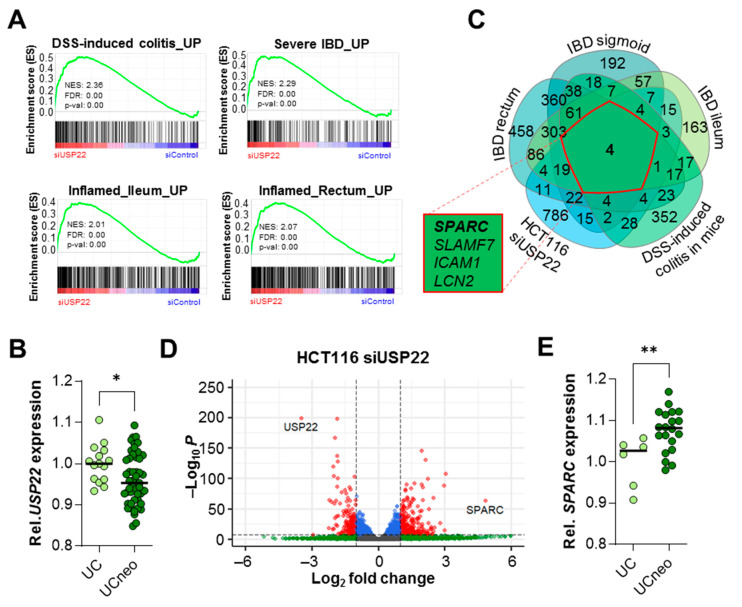
The loss of *Usp22* drives inflammatory bowel disease (IBD)-associated transcriptome-wide changes and promotes *SPARC* expression. (**A**) Gene set enrichment analysis of mRNA-seq data from USP22-depleted HCT116 cells showing enrichment of gene expression signatures upregulated in mice experiencing acute DSS-induced colitis [20] and in IBD patients [21,22]. (**B**) Compared to patients with ulcerative colitis (UC), individuals with UC-associated neoplasm (UCneo) displayed lower *USP22* expression, as demonstrated using microarray analysis [26]. (**C**) Genes upregulated in USP22-depleted HCT116 cells were compared to genes upregulated in IBD patients [21] and mice experiencing colitis [20]. All five gene sets displayed elevated expression of *SPARC*. (**D**) Volcano plot displaying profound *SPARC* upregulation following USP22 depletion in HCT116 cells, as detected using mRNA-seq. (**E**) Patients with UC-associated neoplasm and reduced *USP22* expression showed elevated *SPARC* mRNA levels compared to UC patients [26]. Mean ± SEM. Student’s *t*-test. * *p* ≤ 0.05, ** *p* ≤ 0.01.

To gain insight into the molecular mechanisms by which *Usp22* deficiency influences inflammation and inflammation-driven CRC, we interrogated our previously published mRNA-seq data in USP22-depleted HCT116 cells. In agreement with our in vivo observations, gene set enrichment analysis (GSEA) [18,19] revealed that USP22 depletion resulted in increased expression of genes upregulated in inflamed tissue in DSS-treated mice [20] as well as in patients with IBD [21,22] (Figure 5A). Consistently, *USP22* mRNA levels were decreased in ulcerative colitis (UC) patients displaying neoplasm (UCneo) compared to patients with colitis alone [26] (Figure 5B). When overlapping genes which were upregulated in humans and mice undergoing intestinal inflammation and genes induced in *USP22*-depleted HCT116 cells, we detected only four commonly upregulated genes. One of these was secreted protein acidic and cysteine rich (*SPARC*). Notably, deletion of *Sparc* significantly reduced inflammation-associated signs in DSS-treated mice [27]. Notably, in our HCT116 dataset, *SPARC* was the gene with the highest statistically significant induction (Figure 5D). Consistently, in the UCneo cohort displaying a decrease in average *USP22* expression, an increase in *SPARC* levels was detected (Figure 5E). Together, these results support a role for USP22 in suppressing IBD-associated gene expression signatures, possibly by suppressing the expression of the inflammation mediator *SPARC*.

### 3.6. USP22 Affects H3K27ac and H2Bub1 Occupancy on the SPARC Gene and Upregulates SPARC Levels In Vivo

**Figure 6:**
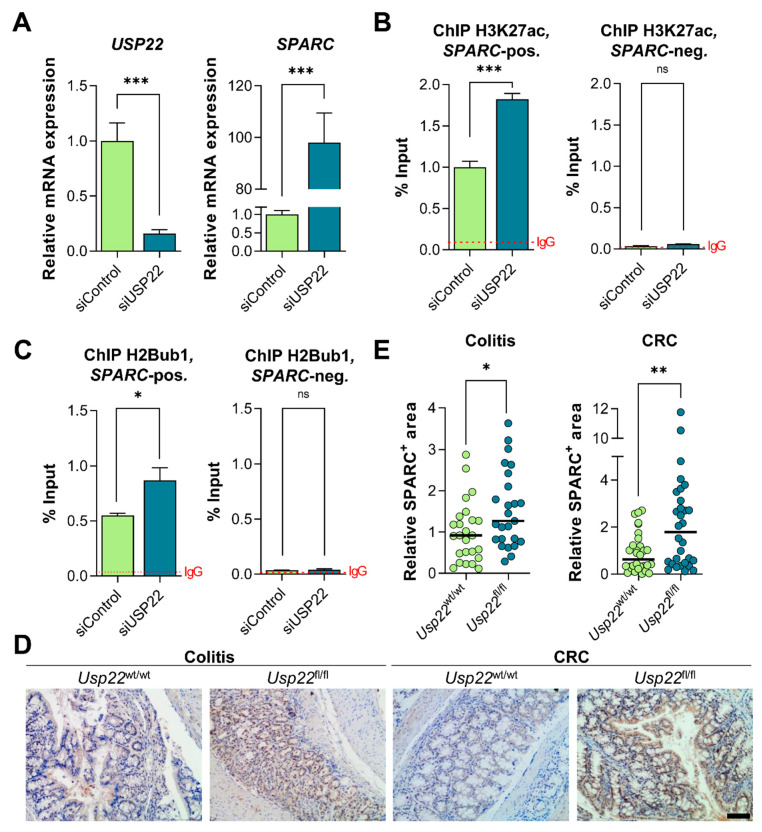
USP22 epigenetically suppresses SPARC expression by affecting the occupancy of acetylated lysine 27 of histone H3 (H3K27ac) and monoubiquitinated histone H2B (H2Bub1). (**A**) The siRNA-mediated reduction of *USP22* levels as well as the induction of *SPARC* expression in HCT116 cells was confirmed using quantitative real-time polymerase chain reaction (qRT-PCR). (**B**) Chromatin immunoprecipitation and subsequent quantitative polymerase chain reaction (ChIP-qPCR) revealed that H3K27ac and (**C**) H2Bub1 occupancy on the *SPARC* gene was significantly increased upon depletion of USP22 in HCT116 cells (*n* = 3). Adjacent H3K27ac- and H2Bub1-negative sites were analyzed as controls. The average signal for the negative control IgG is represented by dotted lines. (**D**) IHC for SPARC in the colon of mice experiencing acute colitis of inflammation-associated CRC revealed an increase in SPARC-positive areas (**E**), as quantified using FIJI. Scale bar: 100 µm. Mean ± SEM, Student’s *t*-test. * *p* ≤ 0.05, ** *p* ≤ 0.01, *** *p* ≤ 0.001.

To confirm the regulation of *SPARC* in our mRNA-seq dataset, we performed qRT-PCR of USP22-depleted HCT116 cells and, as expected, the knockdown resulted in a profound increase in *SPARC* mRNA levels (Figure 6A). Consistent with its strong association with active gene expression, H3K27ac occupancy increased near the transcription start site (TSS) of the *SPARC* gene following USP22 depletion (Figure 6B). Given its reported function as the catalytic subunit of the SAGA deubiquitination module, we also examined H2Bub1 levels in the transcribed region of the *SPARC* gene as well. Notably, USP22 depletion also resulted in increased H2Bub1 occupancy on the *SPARC* gene body (Figure 6C) while global H2Bub1 levels were not increased (Figure S1). Finally, to confirm our findings in vivo, we performed immunohistochemical staining for SPARC on colon sections of Villin-CreER^T2^, *Usp22*^flox^ mice experiencing acute colitis as well as *Apc*^1638N/+^, Villin-CreER^T2^, *Usp22*^flox^ animals with inflammation-associated CRC. Indeed, the intestinal deletion of *Usp22* was associated with higher numbers of SPARC-positive areas in both disease models (Figure 6D,E). Together, USP22 deficiency induced the upregulation of *SPARC* via affecting H3K27ac and H2Bub1 occupancy in human CRC cells and elevated SPARC levels in the colons of mice experiencing colitis and inflammation-associated CRC.

## 4. Discussion

The tumor-promoting function of USP22 has been reported in various malignant diseases. Based on these findings, USP22 is being intensively investigated as a potential therapeutic target, with work ongoing to generate and optimize USP22 inhibitors. However, we and others have demonstrated that USP22 can exert tumor-suppressive functions, putting the benefit of USP22 inhibition into question [8,13,14]. As discussed earlier, several studies suggesting a tumor-promoting function of USP22 did not include appropriate controls and utilized antibodies which were likely to be cross-reactive [8]. These findings suggest that USP22 can act as a tumor-promoting or -suppressive factor in malignant diseases in a context-dependent manner. Further research is needed to clarify the multifaceted role of USP22 in cancer and, importantly, to identify which tumor types or subgroups will benefit from USP22 inhibition.

Interestingly, besides an increased CRC burden, we observed inflammation-associated symptoms as well as epithelial damage in the colon upon the conditional, intestine-specific deletion of *Usp22* in *Apc*-mutated mice [13]. Therefore, we aimed to evaluate the function of USP22 in colorectal inflammation as well as inflammation-associated CRC. In agreement with our previous observations, the intestinal deletion of *Usp22* significantly elevated the disease activity index as well as the degree of epithelial damage in DSS-mediated colitis. In addition, simultaneous *Apc* mutation and intestinal *Usp22* knockout in a model for inflammation-associated CRC resulted in decreased survival and increased colorectal tumor burden. Using a transcriptome-wide approach, we identified *SPARC* as the most highly upregulated gene following USP22 depletion and confirmed these findings in *Usp22*^fl/fl^ mice experiencing acute colitis and CRC.

SPARC is an matricellular glycoprotein which mediates cell–matrix interactions and is involved in various biological processes, including wound repair and tissue remodeling [28,29]. Besides its involvement in cancer [30,31,32], the function of SPARC in inflammation has been demonstrated in earlier studies. In 2007, it was reported that the global ablation of *Sparc* resulted in a reduced renal inflammatory response to angiotensin II-associated hypertension [33]. In a later study, *Sparc* null mice were treated with 3% DSS for seven consecutive days to evaluate the role of SPARC in colitis [27]. Notably, *Sparc* deficiency resulted in a protective effect (i.e., lower pro-inflammatory cytokine production, less epithelial damage), early recovery and a change in the identity of infiltrating cells (fewer infiltrating inflammatory cells and more regulatory T cells). These findings are in agreement with our current study where we identified *SPARC* as a repressed target of USP22, and it is unique in that it was commonly regulated across multiple publicly available datasets analyzing gene expression in colon samples isolated from mice and patients with intestinal inflammation.

In support of increased *SPARC* mRNA levels, we detected elevated occupancy of the active histone marks H3K27ac and H2Bub1 at the transcription start site and gene body, respectively, of *SPARC* in USP22-deficient HCT116 cells. In general, H2Bub1 appears to function by sterically opening chromatin [34] and facilitating transcriptional elongation by promoting the recruitment of the facilitates chromatin transcription (FACT) complex [35]. To date, multiple studies have reported a deubiquitinating activity of USP22 towards H2B [11,36,37]. However, there has been a general discrepancy between the transcription-activating activity of USP22 and the SAGA complex, in general, in promoting transcription and the addition of the H3K9ac-activating histone modification, and its role in deubiquitinating H2Bub1, another histone modification tightly associated with active gene transcription. Our previous and current work suggest that USP22 can serve to either activate gene transcription by promoting SAGA-mediated H3K9 acetylation (i.e., at the *PRKAA2* and *HSP90AB1* genes [13,15]) or repress transcription by deubiquitinating H2B (i.e., at the *SPARC* gene). Thus, future studies addressing these different activities of USP22 and SAGA complex function will be important for the development of strategies targeting this complex.

## 5. Conclusions

Our results reveal a previously unknown function of USP22 in suppressing intestinal inflammation as well as inflammation-associated CRC. These findings significantly expand upon our earlier identification of the tumor suppressor function of USP22 in colorectal cancer. Interestingly, these results mechanistically connect USP22-mediated repression of *SPARC* expression via H2Bub1 deubiquitination, thereby possibly preventing the pro-inflammatory effect of SPARC in DSS-mediated colitis in mice. Further work will be necessary to determine the extent to which the USP22/SPARC axis contributes to the onset and progression of IBD or inflammation-associated CRC in patients.

## Data Availability

The mRNA-seq data presented in this study are openly available at ArrayExpress, accession number: E-MTAB-7393.

## Supplementary Materials

The following are available online at https://www.mdpi.com/article/10.3390/cancers13081817/s1, Figure S1. USP22 depletion did not increase global H2Bub1 levels in HCT116 cells.
