# Supplementary material for: USP22 Suppresses SPARC Expression in Acute Colitis and Inflammation-Associated Colorectal Cancer
Source: Cancers (Basel). 2021 Apr 10;13(8):1817. doi: 10.3390/cancers13081817 (PMC8070211; doi:10.3390/cancers13081817)
Supplement: Supplementary file 1 [file cancers-13-01817-s001.pdf]

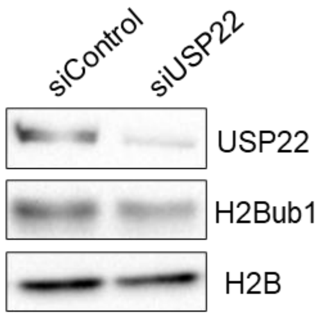

Supplementary Figure S1. USP22 depletion did not increase global H2Bub1 levels in HCT116 cells. Protein was isolated and analyzed by western blot 72 h after siRNA-mediated depletion of USP22 in HCT116 cells. The knockdown of USP22 did not result in increased global H2Bub1 levels.
